# Primary and Secondary Syphilis — United States, 2005–2013

**Published:** 2014-05-09

**Authors:** Monica E. Patton, John R. Su, Robert Nelson, Hillard Weinstock

**Affiliations:** 1 EIS officer, CDC; 2 Division of Sexually Transmitted Disease Prevention, National Center for HIV/AIDS, Viral Hepatitis, STD, and TB Prevention, CDC

In 2013, based on data reported as of April 28, 2014, the rate of reported primary and secondary syphilis in the United States was 5.3 cases per 100,000 population, more than double the lowest-ever rate of 2.1 in 2000. To characterize the recent epidemiology of syphilis in the United States, CDC analyzed data from the National Notifiable Diseases Surveillance System (NNDSS) for cases of primary and secondary syphilis diagnosed during 2005–2013 with a focus on states that reported the sex of sex partners during 2009–2012 to describe reported syphilis among gay, bisexual, and other men who have sex with men (collectively referred to as MSM). During 2005–2013, primary and secondary syphilis rates increased among men of all ages and races/ethnicities across all regions of the United States. Recent years have shown an accelerated increase in the number of cases, with the largest increases occurring among MSM. Among women, rates increased during 2005–2008 and decreased during 2009–2013, with different trends among different racial/ethnic groups. Racial/ethnic disparities in reported syphilis persisted during 2005–2013, likely reflecting social determinants of health, such as socioeconomic status, that might contribute to the burden of syphilis in a community (1). These findings underscore the need for continued syphilis prevention measures among MSM.

CDC analyzed notifiable disease surveillance data on reported syphilis, including patient demographics and stage of syphilis (i.e., primary and secondary, early latent, late, late latent, and congenital*) reported by health departments to NNDSS nationwide for cases diagnosed during 2005–2013. Trends in annual primary and secondary syphilis (representing more recently acquired infections, which usually are infectious) were analyzed for 2005–2013. Geographic trends were analyzed by U.S. census region, age group, and NNDSS racial/ethnic categories (white, black, Hispanic, Asian/Pacific Islander, and American Indian/Alaska Native, for which all racial groups were non-Hispanic). In addition, to describe syphilis among MSM, annual cases of primary and secondary syphilis among MSM that were reported during 2009–2012 were reviewed from 34 states and the District of Columbia, where the sex of sex partners was reported for ≥70% of male primary and secondary syphilis cases (CDC began collecting data on the sex of sex partners in 2005). Rates were calculated using population denominators from the U.S. Census Bureau.

During 2005–2013, the number of primary and secondary syphilis cases reported each year in the United States nearly doubled, from 8,724 to 16,663; the annual rate increased from 2.9 to 5.3 cases per 100,000 population (Table). Men contributed an increasing proportion of cases, accounting for 91.1% of all primary and secondary syphilis cases in 2013. The rate among men increased from 5.1 in 2005 to 9.8 in 2013 (Figure 1). Increases occurred among men of all ages and races/ethnicities, but race/ethnicity shifts occurred in 2009. During 2005**–**2009, rate increases were greatest among black men (104.1%, from 14.6 in 2005 to 29.8 in 2009) compared with Hispanic men (52.0%, 5.0 to 7.6) and white men (19.4%, 3.1 to 3.7). During 2009**–**2013, rates increased among Hispanic men (52.6%, from 7.6 in 2009 to 11.6 in 2013) and white men (45.9%, 3.7 to 5.4), but decreased slightly among black men (6.4%, 29.8 to 27.9). From 2005 to 2009, men aged 20**–**24 years had the greatest percentage increase (149.4%, 8.1 to 20.2), and from 2009 to 2013, men aged 25–29 years (the same approximate birth cohort) had the greatest increase (48.4%, 18.2 to 27.0) (Table).

In 2012, primary and secondary syphilis cases in the 35 reporting areas that reported the sex of sex partners for ≥70% of male cases comprised 83.7% (13,113) of all nationwide cases. In those areas, the proportion of male primary and secondary syphilis cases attributed to MSM increased from 77.0% (6,366) in 2009 to 83.9% (8,701) in 2012. Increases in incidence occurred among MSM of all ages and races/ethnicities from all regions. The greatest percentage increases in cases occurred among Hispanics (53.4%, from 1,291 in 2009 to 1,980 in 2012) and whites (38.1%, 2,449 to 3,381), when compared with blacks (21.2%, 2,267 to 2,747) (Figure 2). By age group, the greatest percentage increases occurred among MSM aged 25**–**29 (53.2%, 1,073 to 1,644).

Among women, the reported primary and secondary syphilis rate increased from 0.9 to 1.5 per 100,000 population per year during 2005**–**2008 and decreased to 0.9 in 2013. This trend occurred among women in all age groups. Rates among white and Hispanic women remained stable; the trend among all women mostly reflected changes in rates among black women (from 4.2 to 7.9 during 2005**–**2009, decreasing to 4.0 in 2013) (Figure 1).

Racial/ethnic disparities in syphilis persisted. In 2013, the primary and secondary syphilis rate among black men was 5.2 times that among white men (27.9 versus 5.4 cases per 100,000 population); the rate among black women was 13.3 times that among white women (4.0 versus 0.3). The rate among Hispanic men was 2.1 times that among white men (11.6 versus 5.4), and the rate among Hispanic women was 2.7 times that among white women (0.8 versus 0.3). These disparities were similar to disparities observed in 2005 (Table).

Across all four U.S. Census regions, primary and secondary syphilis rates were greater in 2013 than in 2005. In 2013, the highest overall regional rate (6.5 cases per 100,000 population) was in the West region. In 2013, for the first time in at least 50 years, the South did not have the highest overall syphilis rate among regions. Regional trends among men and women by race/ethnicity mirrored national trends except in the West region, where there was no decrease among black men during 2009**–**2013. Among women of all races/ethnicities in the West region, rates declined during 2005**–**2010 and increased during 2011**–**2013 (Table).

## Discussion

After being on the verge of elimination in 2000 in the United States, syphilis cases have rebounded. Rates of primary and secondary syphilis continued to increase overall during 2005**–**2013; although rates stabilized during 2009**–**2010, rates have increased since 2011. Increases have occurred primarily among men, and particularly among MSM, who contributed the vast majority of male primary and secondary syphilis cases during 2009**–**2012.

The epidemiology of syphilis among men, including MSM, has shifted since 2009, with larger increases occurring among Hispanic and white men. Despite this increase, disparities in primary and secondary syphilis between black men and other racial/ethnic groups remain large. Many barriers to contacting and treating sex partners exist, including delays in reporting cases to the health department, anonymous partners, physicians who rely on patients to notify their partners (2), and the observed tendency of MSM to notify a smaller proportion of their sex partners than do heterosexuals (3).

These analyses indicate that syphilis prevention measures for MSM of all races/ethnicities need to be strengthened throughout the United States. This could be accomplished by working with private health-care providers because a substantial number of primary and secondary syphilis cases among MSM are reported by private physicians (1). Further, both private and public providers should be aware of the resurgence in syphilis and should be able to recognize the signs and symptoms of syphilis, conduct risk assessments, and screen all sexually active MSM for syphilis at least annually with syphilis serologic tests with confirmatory testing where indicated (4). More frequent screening (i.e., at 3–6 month intervals) is recommended for MSM who have multiple or anonymous sex partners. Disclosure of sexual practices remains difficult for some MSM (5); therefore, providers are encouraged to elicit sexual histories of their patients in a culturally appropriate manner, including recognition of sexual orientation, gender identity, and the sex of patients’ sex partners. Additional resources and training for accomplishing this are available online.†

The increase in syphilis among MSM is a major public health concern, particularly because syphilis and the behaviors associated with acquiring it increase the likelihood of acquiring and transmitting human immunodeficiency virus (HIV) (6). There are reported rates of 50%**–**70% HIV coinfection among MSM infected with primary and secondary syphilis (7) and high HIV seroconversion rates following primary and secondary syphilis infection (8). The resurgence of syphilis, coupled with its strong link with HIV, underscores the need for programs and providers to 1) urge safer sexual practices (e.g., reduce the number of sex partners, use latex condoms, and have a long-term mutually monogamous relationship with a partner who has negative test results for sexually transmitted diseases); 2) promote syphilis awareness and screening as well as appropriate screening for gonorrhea, chlamydia, and HIV infection; and 3) notify and treat sex partners.

What is already known on this topic?

Rates of reported primary and secondary syphilis in the United States have increased since reaching historic lows in 2000. Cases of primary and secondary syphilis increasingly are among males, particularly men who have sex with men (MSM).

What is added by this report?

Primary and secondary syphilis rates increased among men of all ages and races/ethnicities during 2005**–**2013, from 5.1 cases per 100,000 population in 2005 to 9.8 in 2013, when men accounted for 91.1% of all cases reported in the United States. Although rates remain highest among black men (28.1), recent increases were greatest among Hispanic and white men. Currently, syphilis is predominantly an MSM epidemic.

What are the implications for public health practice?

Syphilis prevention measures for MSM of all races/ethnicities should be strengthened throughout the United States, including 1) encouraging safer sexual practices (e.g., reducing the number of sex partners, using latex condoms, and having a long-term mutually monogamous relationship with a partner who has negative test results for sexually transmitted diseases); 2) promoting syphilis awareness and screening as well as appropriate screening for gonorrhea, chlamydia, and human immunodeficiency virus infection; and 3) notifying and treating sex partners. In addition, efforts to prevent and treat syphilis among heterosexual men and women should continue in order to prevent congenital syphilis.

Public health officials should seek to improve the quality of data regarding the sex of sex partners, share local MSM, sexually transmitted disease, and HIV data consistent with local laws and regulations with medical providers to increase their awareness of disease burden in their communities, and ensure that providers can recognize syphilis symptoms. Two CDC cooperative agreements are encouraging local and state participants to make MSM a priority population and direct resources to areas of greatest need based on local epidemiology (9,10). CDC, in collaboration with state and local partners, health-care providers, and MSM-oriented organizations, is also engaged in research to better understand risk factors for syphilis among MSM, develop improved care models to better reach and serve MSM populations, assess whether MSM are being tested and treated appropriately, and determine what barriers exist in the diagnosis and treatment of syphilis among MSM.

The continued decline of primary and secondary syphilis rates among black women since 2008 is encouraging and might suggest that targeted efforts to reduce syphilis among certain populations have had some success. Although primary and secondary syphilis is currently a predominantly MSM epidemic, it is important that efforts to prevent syphilis among heterosexual men and women continue, especially given the severe consequences of syphilis infection acquired in utero, including stillbirths.

The findings in this report are subject to at least two limitations. First, primary and secondary syphilis case-report data likely underestimate the true number of syphilis infections in the United States because of underreporting of diagnosed cases, infected persons not accessing health care, misdiagnosis, and the fact that primary and secondary syphilis cases amounted to only 31.4% of all syphilis cases reported in 2012. Second, the findings for MSM included only data from 34 states and the District of Columbia, where the sex of sex partners was reported for 70% or more of male primary and secondary syphilis cases. For 12% of cases in these 35 reporting areas, the sex of sex partners was unknown.

Despite decreasing rates of primary and secondary syphilis in the late 1990s in the United States, the resurgence of cases in recent years highlights the fact that challenges remain, and the increases among MSM are particularly concerning. Public health practitioners might want to consider focusing on efforts to strengthen linkages with practicing physicians to improve case identification and reporting, partner-notification programs, and outreach to MSM.

## Figures and Tables

**FIGURE 1:**
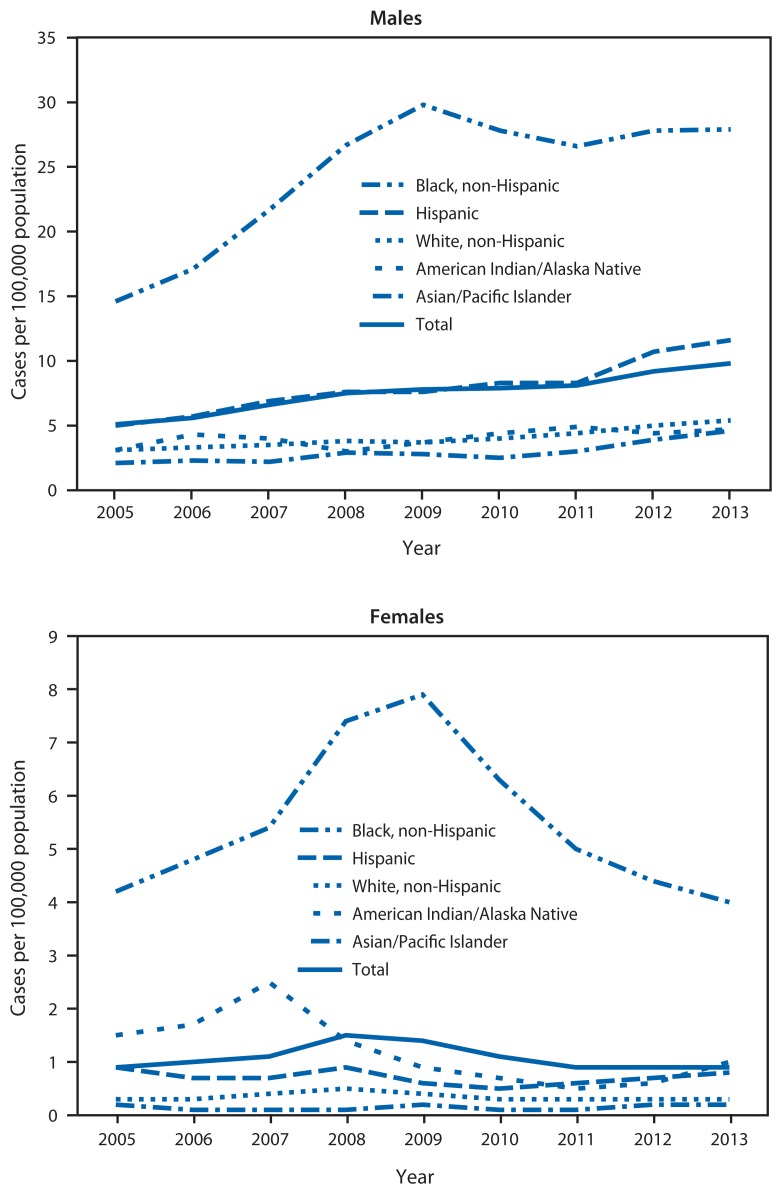
Annual rate of primary and secondary syphilis cases among males and females, by race/ethnicity — National Notifiable Diseases Surveillance System, United States, 2005–2013

**FIGURE 2:**
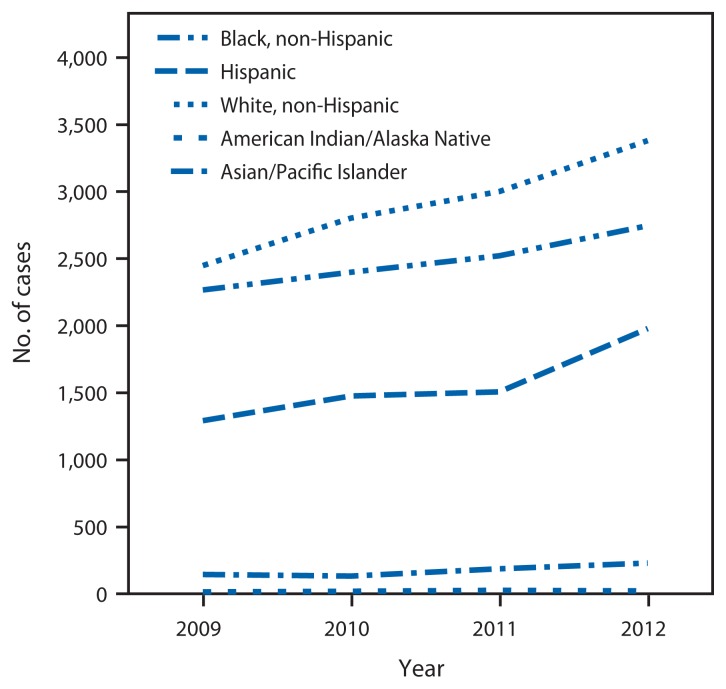
Number of primary and secondary syphilis cases among men who have sex with men, by race/ethnicity — National Notifiable Diseases Surveillance System, 34 states* and the District of Columbia with complete sex partner data,^†^ 2009–2012 * Arkansas, California, Colorado, Connecticut, Florida, Hawaii, Idaho, Illinois, Indiana, Iowa, Kansas, Kentucky, Maine, Maryland, Massachusetts, Michigan, Minnesota, Missouri, Nevada, New Hampshire, New Jersey, New York, Ohio, Oklahoma, Pennsylvania, Rhode Island, South Carolina, South Dakota, Tennessee, Texas, Vermont, Virginia, Washington, and West Virginia. ^†^ Sex of partners reported for ≥70% of cases of primary and secondary syphilis in males aged ≥15 years during 2009–2012.

**TABLE:** Number and rate* of primary and secondary syphilis cases, by race/ethnicity, U.S. Census region,† age group, and sex — National Notifiable Diseases Surveillance System, United States, 2005–2013

	2005		2006		2007		2008		2009		2010		2011		2012		2013§	
									
Characteristic	No.	Rate	No.	Rate	No.	Rate	No.	Rate	No.	Rate	No.	Rate	No.	Rate	No.	Rate	No.	Rate
**Males**																		
**Race/ Ethnicity**																		
White, non-Hispanic	3,049	3.1	3,249	3.3	3,456	3.5	3,789	3.8	3,702	3.7	3,973	4.0	4,321	4.4	4,931	5.0	5,293	5.4
Black, non-Hispanic	2,607	14.6	3,086	17.1	3,952	21.7	4,876	26.7	5,535	29.8	5,236	27.8	5,074	26.6	5,369	27.8	5,383	27.9
Hispanic	1,111	5.0	1,304	5.7	1,627	6.9	1,844	7.6	1,904	7.6	2,119	8.3	2,182	8.3	2,889	10.7	3,118	11.6
A/PI	136	2.1	154	2.3	155	2.2	201	2.9	203	2.8	190	2.5	139	3.0	318	3.9	381	4.6
AI/AN	37	3.1	53	4.3	49	4.0	38	3.0	47	3.7	55	4.4	63	4.9	57	4.4	61	4.7
**Region**																		
Northeast	1,171	4.4	1,314	4.9	1,741	6.5	1,924	7.2	1,946	7.2	2,068	7.7	1,987	7.4	2,292	8.4	2,466	9.1
Midwest	1,059	3.3	1,020	3.1	1,113	3.4	1,485	4.5	1,638	5.0	1,944	5.9	1,926	5.8	1,987	6.0	2,311	7.0
South	3,170	6.0	3,619	6.8	4,378	8.1	5,008	9.1	5,657	10.2	5,029	9.0	5,164	9.1	5,904	10.3	5,933	10.3
West	1,983	5.8	2,340	6.7	2,537	7.2	2,838	8.0	2,523	7.0	2,940	8.2	3,376	9.3	4,007	10.9	4,465	12.2
**Age group (yrs)**¶																		
15–19	251	2.3	332	3.0	416	3.8	585	5.3	661	6.0	617	5.5	606	5.5	640	5.8	663	6.0
20–24	875	8.1	1,080	9.9	1,461	13.5	1,877	17.3	2,242	20.2	2,429	22.1	2,582	22.8	2,859	24.8	3,042	26.3
25–29	1,007	9.8	1,330	12.6	1,574	14.6	1,851	16.9	2,027	18.2	2,131	20.0	2,277	21.2	2,641	24.4	2,925	27.0
30–34	1,178	11.6	1,056	10.6	1,303	13.2	1,489	15.0	1,571	15.5	1,597	16.0	1,657	16.1	2,023	19.3	2,179	20.8
35–39	1,394	13.2	1,426	13.4	1,529	14.4	1,568	14.8	1,409	13.6	1,313	13.1	1,265	13.0	1,443	14.9	1,597	16.4
40–44	1,253	11.0	1,362	12.2	1,551	14.1	1,573	14.6	1,476	14.1	1,448	13.9	1,408	13.5	1,544	14.8	1,515	14.5
45–54	1,080	5.2	1,277	6.0	1,463	6.8	1,790	8.2	1,815	8.3	1,877	8.5	1,999	9.1	2,310	10.6	1,398	11.0
55–64	283	1.9	340	2.2	379	2.4	412	2.5	475	2.8	457	2.6	510	2.8	586	3.2	682	3.7
≥65	59	0.4	87	0.6	86	0.5	102	0.6	84	0.5	105	0.6	137	0.8	138	0.7	159	0.8
**Total**	**7,383**	**5.1**	**8,293**	**5.6**	**9,769**	**6.6**	**11,255**	**7.5**	**11,764**	**7.8**	**11,981**	**7.9**	**12,453**	**8.1**	**14,190**	**9.2**	**15,175**	**9.8**
**Females**																		
**Race/ Ethnicity**																		
White, non-Hispanic	263	0.3	295	0.3	370	0.4	474	0.5	418	0.4	299	0.3	261	0.3	274	0.3	293	0.3
Black, non-Hispanic	828	4.2	942	4.8	1,075	5.4	1,478	7.4	1,605	7.9	1,296	6.3	1,041	5.0	931	4.4	852	4.0
Hispanic	183	0.9	158	0.7	163	0.7	209	0.9	144	0.6	118	0.5	142	0.6	193	0.7	209	0.8
A/PI	11	0.2	10	0.1	7	0.1	9	0.1	15	0.2	11	0.1	12	0.1	14	0.2	21	0.2
AI/AN	19	1.5	22	1.7	32	2.5	18	1.4	12	0.9	9	0.7	6	0.5	8	0.6	13	1.0
**Region**																		
Northeast	91	0.3	86	0.3	96	0.3	100	0.4	130	0.5	125	0.4	116	0.4	125	0.4	115	0.4
Midwest	137	0.4	156	0.5	147	0.4	237	0.7	212	0.6	309	0.9	249	0.7	251	0.7	239	0.7
South	884	1.6	984	1.8	1,228	2.2	1,697	3.0	1,756	3.0	1,225	2.1	997	1.7	915	1.5	818	1.4
West	227	0.7	232	0.7	221	0.6	208	0.6	134	0.4	121	0.3	139	0.4	167	0.5	299	0.8
**Age group (yrs)**¶																		
15–19	192	1.9	233	2.2	248	2.4	318	3.0	344	3.3	313	2.9	258	2.5	238	2.3	202	1.9
20–24	305	3.0	299	2.9	356	3.5	520	5.1	570	5.5	474	4.5	403	3.7	417	3.8	429	3.9
25–29	205	2.1	241	2.4	265	2.6	404	3.9	377	3.6	322	3.1	268	2.5	266	2.5	272	2.6
30–34	150	1.5	163	1.7	193	2.0	244	2.5	286	2.9	197	2.0	187	1.8	182	1.7	164	1.6
35–39	179	1.7	154	1.5	191	1.8	241	2.3	203	2.0	140	1.4	115	1.2	120	1.2	121	1.2
40–44	164	1.4	153	1.4	192	1.7	202	1.9	167	1.6	104	1.0	91	0.9	70	0.7	101	1.0
45–54	111	0.5	165	0.8	200	0.9	236	1.0	218	1.0	176	0.8	120	0.5	128	0.6	122	0.5
55–64	20	0.1	35	0.2	30	0.2	46	0.3	42	0.2	36	0.2	43	0.2	27	0.1	36	0.2
≥65	5	0.0	2	0.0	9	0.0	9	0.0	8	0.0	6	0.0	3	0.0	5	0.0	12	0.0
**Total**	**1,339**	**0.9**	**1,458**	**1.0**	**1,692**	**1.1**	**2,242**	**1.5**	**2,232**	**1.4**	**1,780**	**1.1**	**1,501**	**0.9**	**1,458**	**0.9**	**1,471**	**0.9**
**Overall total** **	**8,724**	**2.9**	**9,756**	**3.3**	**11,466**	**3.8**	**13,500**	**4.4**	**13,997**	**4.6**	**13,774**	**4.5**	**13,970**	**4.5**	**15,667**	**5.0**	**16,663**	**5.3**

**Abbreviations:** A/PI = Asian/Pacific Islander; AI/AN = American Indian/Alaska Native.

* Per 100,000 population.

† *Northeast:* Connecticut, Maine, Massachusetts, New Hampshire, New Jersey, New York, Pennsylvania, Rhode Island, and Vermont; *Midwest:* Illinois, Indiana, Iowa, Kansas, Michigan, Minnesota, Missouri, Nebraska, North Dakota, Ohio, South Dakota, and Wisconsin; *South:* Alabama, Arkansas, Delaware, District of Columbia, Florida, Georgia, Kentucky, Louisiana, Maryland, Mississippi, North Carolina, Oklahoma, South Carolina, Tennessee, Texas, Virginia, and West Virginia; *West:* Alaska, Arizona, California, Colorado, Hawaii, Idaho, Montana, Nevada, New Mexico, Oregon, Utah, Washington, Wyoming.

§ Data are as of April 28, 2014.

¶ Includes persons aged ≥15 years.

** Cases among persons aged ≤14 years not shown.

## Footnotes

* Additional information available at http://wwwn.cdc.gov/nndss/script/casedef.aspx?condyrid=941&datepub=1/1/2014.

† Available at http://www2a.cdc.gov/stdtraining/self-study/syphilis/default.htm, http://www.cdc.gov/std/syphilis/stdfact-msm-syphilis.htm, and http://nnptc.org/resourcetags/sexual-history.
